# “What other choices might I have made?”: Sexual Minority Men, the PrEP Cascade and the Shifting Subjective Dimensions of HIV Risk

**DOI:** 10.1177/10497323221092701

**Published:** 2022-05-26

**Authors:** Mark Gaspar, Alex Wells, Mark Hull, Darrell H. S. Tan, Nathan Lachowsky, Daniel Grace

**Affiliations:** 1 Dalla Lana School of Public Health, University of Toronto, Toronto, ON, Canada; 2 University of Victoria, Victoria, BC, Canada; 3 University of British Columbia, Vancouver, BC, Canada; 4 St. Michael’s Hospital and University of Toronto, Toronto, ON, Canada

**Keywords:** HIV prevention, pre-exposure prophylaxis, PrEP Cascade, gay, bisexual, queer men

## Abstract

The PrEP Cascade is a dominant framework for investigating barriers to HIV pre-exposure prophylaxis (PrEP), an HIV prevention tool. We interviewed 37 PrEP users and 8 non-PrEP users in Ontario and British Columbia, Canada, about their decision-making through the Cascade. Participants were HIV-negative gay, bisexual, and queer men (GBQM). The data were analyzed using thematic analysis. PrEP decision-making was based on pragmatic considerations (logistics, costs, and systemic barriers), biomedical considerations (efficacy, side-effects, and sexually transmitted infections), and subjective considerations (identity, politics, and changing sexual preferences). Affective attachments to established versions of “safer sex” (condoms and serosorting) made some GBQM less likely to try PrEP. Some GBQM expressed increased social expectations to use PrEP, have condomless sex, and serodifferent sex. These findings support offering PrEP at no-cost, offering individualized counseling and community-based opportunities to discuss PrEP use and changing sexual practices, and improving communication on the manageability of PrEP side-effects.

## Introduction

In 2016, Health Canada approved the daily use of tenofovir disoproxil fumarate with emtricitabine (TDF/FTC) by HIV-negative individuals as pre-exposure prophylaxis (PrEP), a highly effective HIV prevention tool. In 2020, Health Canada also approved tenofovir alafenamide with emtricitabine (TAF/FTC) as HIV PrEP. Combined with the confirmation that for those living with HIV, maintaining an undetectable (suppressed) viral load makes HIV untransmittable to sexual partners (“U=U” and the “Treatment as Prevention” model), the advent of PrEP was a major turning point in the HIV epidemic (Philpot et al., 2020). These advances have been associated with a decline in HIV incidence for the first time in decades among gay, bisexual, queer, and other men who have sex with men (GBQM) in some regions like the UK (Gee et al., 2019). In Canada, GBQM still have some of the highest incidence rates in the country, making up 52.2% of new HIV infections in 2018 (Public Health Agency of Canada, 2020).

PrEP implementation has been beset by several challenges in Canada. First, there is its cost. Some provincial health insurance programs like the Medical Service Plan (MSP) in British Columbia (BC) fully cover PrEP for GBQM who meet provincial guidelines. Across Canada, Indigenous people who are covered under the Federal Non-Insured Health Benefits Program can get PrEP as part of their treaty rights. In Ontario, PrEP’s costs are completely covered for individuals under the age of 25, over the age of 65, and on disability insurance or social assistance (Ontario Works). Individuals with lower incomes can also access PrEP through the publicly funded Trillium Drug Benefit Program in Ontario, though co-payments scaled to income are required. Otherwise, a PrEP user must pay out of pocket (approximately $200 CAD a month) or use a private drug insurance policy such as those offered through work or school.

Second, PrEP implementation is limited by the “centralization” of PrEP care (Charest et al., 2021). In Canada, primary care providers less familiar with HIV or sexual minority health may be reluctant to prescribe PrEP, preferring patients to consult with HIV specialists (Charest et al., 2021). Consequently, GBQM living in larger cities like Toronto, Ottawa, or Vancouver, and those who have primary care providers that specialize in sexual health care and HIV, may find it easier to access PrEP than those not linked up to sexual and gender minority friendly care.

Other barriers include stigma toward PrEP users (i.e., that they are “promiscuous” and thus untrustworthy) (Grace et al., 2018; Newman et al., 2018; Philpot et al., 2020), patients’ concerns with efficacy (Williamson et al., 2019), critiques regarding the commodification of the epidemic (Young et al., 2016), worries regarding side-effects, and increased sexually transmitted infections (STIs) due to condomless sex (Philpot et al., 2020). These barriers have led some to conclude that PrEP uptake could be improved, especially among GBQM at the highest risk for HIV (Gaspar et al., 2021; Quinn et al., 2019).

While PrEP should be easy to access for all those who need and want it, PrEP is not a necessary intervention for all GBQM. However, it remains unclear how exactly GBQM are determining whether PrEP is useful or not and whether decisions to not take PrEP are primarily a result of systemic barriers to healthcare, social and cultural factors, a preference for established prevention methods, or because some GBQM do not consider themselves at high enough risk. In Canada, there has been limited qualitative research on how GBQM are navigating PrEP access given the implementation challenges noted above (Newman et al., 2018). Our current study seeks to correct this gap by drawing on interviews conducted with HIV-negative GBQM about their PrEP decision-making, including the choice to initiate, pause, stop, or never try PrEP. Participants included current and former PrEP users, and those who have never tried PrEP. We begin by outlining the theoretical frameworks informing our approach.

## Sexuality in and Through the Cascade

The PrEP Continuum of Care, also known as the PrEP Cascade, is the dominant framework for investigating the efficacy of PrEP programs and barriers to care (Holt et al., 2020). The Cascade is organized as patient decision-making steps and conditions that must be achieved to access PrEP *or* as conditions health providers must meet to get GBQM on PrEP. In this article, we focus on the Cascade from the point of view of PrEP users and potential users. The Cascade is conceptualized differently across studies (Holt et al., 2020), but encapsulates the following: (1) an awareness of being at risk for HIV; (2) an awareness of PrEP and one’s eligibility for PrEP and a willingness to take PrEP; (3) discussing PrEP with a care provider; (4) receiving a PrEP prescription; and (5) getting and adhering to PrEP, including following through with routine testing for STIs, HIV, and monitoring side-effects.

Newman et al. (2018) propose a non-linear view of the Cascade that considers how GBQM tend to stop, pause, and restart PrEP. Sexual risk-taking is rarely static and a GBQM’s need for PrEP is likely to change over time. For example, based on data collected as part of the current study, we previously reported on how many GBQM in Ontario stopped and restarted their PrEP use in response to shifting COVID-19 control measures that affected their ability to have sex with casual or new partners (Gaspar et al., 2022). Newman et al.’s (2018) analysis is influenced by research on HIV and GBQM which acknowledges that people do not always “behave” the way health promotion models would predict. As Holt et al. (2020) state about the PrEP Cascade, “there are tensions between top–down or researcher-driven measures and participant or user perceptions of HIV risk, which may not match” (e17). A social actor’s perceptions of HIV risk are not merely guided by rational, “objective” facts, but are informed by their understandings of what it means to be a moral and responsible sexual citizen in the context of a continually evolving and stigmatized epidemic (Gaspar, 2017; Gaspar et al., 2022; Williamson et al., 2019).

Thus, Newman et al. (2018) argue for the need to examine the role of meaning and identity which inform GBQM’s journeys through the Cascade. Social scientists have observed that safer sex strategies and biomedical advances in HIV can have significant effects on GBQM’s relationships, identity, and on community norms (Gaspar, 2017; García-Iglesias, 2020). Beyond sexual and health behavior, biomedical advances also influence *sexuality* as, in Foucauldian parlance, a biopolitical process that turns desire, pleasure, and bodies into self-regulating subjectivity (Foucault, 1990). PrEP does not just allow one to manage sexual health risks, but ontologically, it changes *what sex is*, and what it means to be a gay, bisexual or queer man at *this* specific moment in the epidemic (Grov et al., 2021; Kippax & Race, 2003; Race, 2014).

Based on research from earlier in the epidemic that examined the interaction between biomedical advances and sexual practice (i.e., such as negotiated safety, the decision to have condomless sex with men who have the same HIV status), Kippax and Race (2003) note that there is a need to explore sexuality and sexual health decision-making as a broader set of *social* practices—as not just individual choices, but as relational, nuanced, and context-driven (Gaspar, 2019). PrEP may represent a newer prevention tool, but its social impact is informed by longer standing historical and sociological dynamics. For example, Hammack et al. (2019) have used the life course model to demonstrate how some older men (i.e., the AIDS-1 generation who were sexually active during the height of the AIDS crisis in the 1980s in North America) and the “post-AIDS generation” (i.e., those who came of sexual age after the AIDS crisis years and who we refer to below as the PrEP and U=U generation as they became sexually active in the 2010s) have been more immediately receptive to PrEP compared to some men from the AIDS-2 generation. The AIDS-2 generation, who were men born in the 1980s and early 90s (millennials), have never known a world without HIV. Though this generation was not sexually active during the AIDS crisis years, they remember the earliest phases of the epidemic through media and potentially through personal relationships. They also had to contend with HIV risk before the biomedical advances of the 2010s. As such, their skepticism to PrEP is related to their sexual identity being symbolically tied to notions of HIV risk, HIV anxiety, stigma (Gaspar, 2017), and to the necessity of condoms (Hammack et al., 2019).

Kippax and Stephenson (2012) argue that the scholarly lines drawn between biomedical and public health research—within which models like the PrEP Cascade fall—and social science approaches to HIV are fallacious. Epidemiological models may be helpful in explaining patterns that can help target health promotion activities. However, these tools can respond more effectively to the needs of GBQM when we have a better understanding of the complexity of sexual health decision-making in the real world—that is, when they take sexuality as a social practice into consideration, an analytic approach best suited to qualitative methods that can assess context (Gaspar, 2019). Thus below, we analyze how GBQM navigate the Cascade from HIV risk assessment, to deciding to start or stop PrEP. However, we do so with attention to the social, cultural, and political factors informing their choices that health providers and health promotion specialists need to be aware of in order to best frame their messaging and care.

## Methods

The following analysis is derived from 45 in-depth, one-on-one interviews conducted between March 2020 and October 2020 in Ontario and BC. These baseline data are part of a longitudinal series where research participants are invited for annual interviews over 3 years. This qualitative study is one component of the mixed-methods implementation science project called PRIMP, which is working to improve PrEP service delivery for GBQM in Ontario and BC. Ethics approval was granted by the University of Toronto, St Michael’s Hospital, the University of Victoria, and the University of British Columbia Clinical Research Ethics Board.

A semi-structured guide was used to organize the interview. Study objectives and questions were developed through collaboration with a community advisory board comprised of stakeholders from GBQM health organizations. The interview guide started with socio-demographic questions, then explored participants’ general health concerns and healthcare access experiences, participants’ PrEP usage history, and HIV risk evaluation. Participants were asked about their specific sexual practices and PrEP experiences, and any changes they were observing within the GBQM community because of PrEP. We also discussed the impact of COVID-19 lockdown procedures on sexual practices and PrEP use, which are reported on elsewhere (Gaspar et al. 2022). A web site and poster were used to recruit PrEP users and non-PrEP users. Recruitment materials were shared with our participating clinics and community partners over social media. Participants were also encouraged to share the study details with their networks. We aimed to recruit a diverse sample factoring in age, race, gender identity, PrEP usage history, and city location in Ontario (Toronto, Ottawa) and BC (Victoria, Vancouver). The men had to identify as GBQM and/or be sexually active with other men.

One interview was conducted in person before the start of COVID-19 lockdowns in Canada. All the remaining interviews were conducted online or over the phone with interviewers who identify as queer. The study team with comprised mostly of people who self-identify as GBQM. The interviewees provided informed consent and received $30 CAD for their participation. The interviews were audio recorded, transcribed verbatim, and analyzed on NVivo software using thematic analysis (Braun & Clarke, 2006).

The coding process included both deductive and inductive phases. The first rounds of coding deductively organized the data into large thematic clusters that corresponded to the key topical areas and research questions covered in the interview guide. From here, the second round of coding was more granular and inductive, and followed Braun and Clarke’s (2006) use of semantic analysis to capture some of the underlying assumptions and ideologies that shaped the participants’ responses. This stage of analysis was iterative, with a consistent return to the literature described above to strengthen the theoretical and conceptual framing, as well as comparing emergent themes to the logic of the PrEP Cascade. Analytic emphasis was placed on understanding the contexts that informed why some people had never used PrEP, why some people had stopped PrEP, and why some people decided to go on PrEP in the first place. We aimed to understand how PrEP decision-making was motivated by standard barriers to healthcare (i.e. cost, side-effect concerns), how varied social and cultural beliefs on sexual practice and identity informed decisions to take PrEP, and how the increased accessibility of PrEP was impacting notions of sexual identity, agency, and community.

## Results

The above table outlines the socio-demographics of the sample (Table 1).

**Table 1.** Socio-Demographic Characteristics

Age	*n* = 45	
20–29	14	31%
30–39	18	40%
40–49	6	13%
>50	7	16%
Race		
White	17	38%
Black	6	13%
East Asian	7	16%
Middle-Eastern	4	9%
South Asian	4	9%
South East Asian	1	2%
Latino	3	7%
Indigeneous	3	7%
Gender		
Cis man	41	91%
Trans man	4	9%
Sexual identity		
Gay	39	87%
Bisexual	4	9%
Queer	1	2%
Pansexual	1	2%
PrEP use		
Taking or have taken PrEP	37	82%
Have never taken PrEP	8	18%
Location		
Ontario: Toronto and the greater Toronto area	17	38%
Ontario: Ottawa	8	18%
British Columbia: Vancouver	15	33%
British Columbia: Victoria	6	13%

Below we explore the complex considerations that informed participants’ *evolving* PrEP decision-making. We categorize these considerations as pragmatic, biomedical, and subjective.

### Pragmatic Considerations: The Work of Access

*Pragmatic considerations* refer to the logistical effort, financial factors, and other systemic barriers to healthcare services that determine ease of access to PrEP. Some participants in both provinces who got PrEP before Health Canada approval in 2016 discussed the initial challenges of getting it, including having their physicians not know what PrEP was or being reluctant to prescribe them PrEP. In some cases, a participant was not “out” as GBQM to their physician and needed to find an alternative health provider. Participants in both provinces acknowledged that BC had a somewhat distinctive and more proactive PrEP prescribing culture compared with Ontario, and that it was easier for people to afford PrEP in BC.

The cost of PrEP remained, *by far*, the biggest barrier reported by participants, particularly in Ontario. One PrEP user described how being unable to afford PrEP before it was covered in BC put him “at a higher risk.” Another PrEP user mentioned that when he first heard about PrEP he thought that it was “only for rich people.” He eventually learned that he had access to PrEP for free because he is Indigenous: “Apparently I was eligible, but no one told us.” A participant living in Ontario discussed stockpiling PrEP in case he lost his job and its associated drug plan. In Ontario, not having private drug insurance prevented some participants from accessing PrEP. As such, some GBQM felt that they had to be at *high* risk for HIV to justify the expense of PrEP. As one man stated, “Honestly, I feel no [I am not going to try PrEP] because I’m trying to save. So, it’s gonna be expensive. Like $100 and I know I’m not at high risk.”

The cost of PrEP also created complex situations for some GBQM as they managed career changes alongside negotiating precarious work or navigating getting onto their partner’s drug plan. Some men who self-reported working in the “gig economy” discussed not having access to the drug insurance necessary to cover PrEP (or any other medications). One PrEP user mentioned that he would have to move for his job and how it would be challenging to pay for PrEP if he no longer lived in BC. A man in Ontario described how stopping PrEP was a factor in his decision-making to start his own company as he would be leaving a job with drug benefits; no longer being able to afford PrEP caused “a bit of anxiety” and a “reduced quality of life.”

Despite these systemic and logistical barriers, the general consensus among participants was that if one wanted PrEP, one could now get it. As one man who had decided not to take PrEP stated, “I think they’re giving it away like candy.” While this somewhat facetious comment may minimize the hassles to access just noted, it demonstrates the perception that getting PrEP has become easier over time and implicitly acknowledges a growing sense that GBQM should, at the very least, consider taking PrEP (more on this below). Nonetheless, PrEP access still required “a bit of work.” Getting PrEP took energy, time, and money (co-payments, transit payments, gas money, rescheduling or taking time off work). Consequently, for each GBQM, the benefits of being on PrEP had to outweigh the continuous effort and costs required to stay on it.

Some participants understood themselves as being at high risk for HIV, *needing* PrEP, and barriers such as cost as prohibiting their access to necessary care. However, PrEP users who found the financial means to initiate PrEP tended to consider any remaining obstacles throughout the Cascade minimal and solvable.

## Biomedical Considerations: Assessing Health and Bodily Effects

The second set of considerations were *biomedical considerations*, an assessment of PrEP’s health and bodily effects. These were made up of concerns with efficacy, side-effects, and STIs. If a GBQM vocalized strong biomedical concerns he tended to be *PrEP hesitant*, accepting PrEP’s benefits for others, but not (yet) interested in pursuing it for himself.

While some participants described initially worrying about PrEP’s efficacy, in general, the sample was now convinced that PrEP was highly effective at preventing HIV. However, side-effects were a significant factor generating PrEP hesitancy, with several mentioning a concern about the impact of PrEP on their kidneys, bones, and liver, which, though rare and reversible, are real potential complications that can arise from sustained PrEP use. Some participants stated that potential long-term and unknown side-effects were why they had chosen not to take PrEP. One man who had decided that he did not want to be on PrEP yet, articulated his apprehensions as such: “I don’t have a concern with [immediate, short-term] side-effects. I just haven’t seen what it does to the body. We have to see what it does to the body after five to ten years, or three years, you know?” One participant who was taking PrEP described “pre-conceptions that there are a lot of side effects” with PrEP, though he understood that PrEP is relatively safe. Another PrEP user also described the side-effects as a “little scary because technically it’s an HIV medication,” a belief that is possibly the legacy of the significant side-effects of early HIV treatments. While one PrEP using interviewee regularly paused his PrEP use due to his worries regarding potential side-effects. However, most participants taking PrEP tended to rationalize short-term side-effects (e.g., nausea) as a normal aspect of taking medication that “reverses and goes back” if you stop taking PrEP. No PrEP user reported experiencing significant side-effects on their kidney, bones or liver.

A concern with STIs also affected PrEP decision-making. If a participant was worried about STIs then he tended to consider condoms necessary with casual partners, rendering PrEP less valuable. For instance, one participant articulated his decision to not take PrEP as such:

Yes, [I am concerned with] side effects and also what’s the . . . if I still need to wear a condom—so a condom is supposed to prevent HIV and other STIs. If you take PrEP it’s going to protect you against HIV but not everything else. So then taking PrEP and using a condom I think is overdoing it. So just use a condom and get it over with. That’s my simple-minded thinking. And I don’t like condoms. So that’s why I would end up with the unsafe sex situation [on PrEP].

In summary, biomedical considerations played a role in informing PrEP decision-making throughout the Cascade, particularly decisions to start or pause use. GBQM weighed the value of PrEP given the availability of other HIV prevention methods (e.g., condoms) and concerns with side-effects. These side-effect related anxieties tended to reflect a broader hesitancy to (newer) medication use more generally, rather than being a PrEP specific worry. However, a minority of men vocalized an explicit concern regarding the toxicity of HIV medications.

## Subjective Considerations: Sexuality and Shifting Social Norms

The participants’ PrEP decision-making was also informed by *subjective considerations*. These refer to the social, cultural, political, and affective dimensions shaping *sexuality* and encapsulate how PrEP (dis)use may change perceptions of the self, identity, and relationships.

### Changes in Sexual Practices and Flexible PrEP Use

Participants discussed how PrEP changed their sexual practices, including having more sex and using condoms less. With PrEP, one participant discussed how he was having sex with a “a considerable amount” of partners and “less than 5% [are] with condoms.” Another interviewee mentioned how with PrEP “my condom use has gone way down,” while another described having “more encounters overall.” PrEP also allowed some participants to explore non-monogamy in their primary relationships. For example, one man on PrEP discussed how his partner would not have been willing to open up their relationship sexually without PrEP.

Conversely, many PrEP users’ sexual practices were described as unchanged. For instance, one interviewee described his sex life as staying “relatively the same” with PrEP. Some PrEP users observed such little change in their sexual practices that they questioned whether PrEP was even warranted, especially considering the logistical effort and costs described above. As one participant vocalized, “like I’m wasting it. Like I’m taking it but I’m still not that [sexually] active, you know?” Another PrEP user articulated how “I was kind of wondering why I started taking it, because I felt like I wasn’t really . . . I really didn’t need it. But now I’m glad that I’ve kept on it.” These reflections complicate the idea of risk perception as a primary driver to PrEP initiation on the Cascade. Some men may start PrEP not because they perceive themselves as being at *high* risk, but from a more ambivalent standpoint of trying it out to see if PrEP may be beneficial—an evaluation process that takes some time as sexual practices shift or not.

Many PrEP users discussed how they stopped, paused, and restarted PrEP. One interviewee mentioned taking “some breaks” and then starting PrEP again when he knew sex was forthcoming. Another discussed taking PrEP “on-demand” (i.e., taking PrEP 1 day before, the day of and the day after a sexual encounter, instead of everyday) because he was only having sex once a month, so “that way if I’m taking something I’m not needing and I don’t have to take every day. So, it feels better to be able to take it on-demand than [continuously].” One man described how his sex life was “situational” and how sometimes he would stop taking PrEP because he did not have any time to meet sexual partners. Having sex again required him to plan ahead: “I guess even if someone asks me, oh do you want to have sex and I say yes, if I’m not on PrEP at that time I’ll usually kind of set a date with them a week from now or more than a week so I have time to ramp up the PrEP again.” However, this participant said that it was difficult to find reliable information about taking PrEP episodically. Many other participants did not know about taking PrEP episodically or on-demand.

One interviewee discussed his plans to stop using PrEP once he turned 25 and was no longer covered by provincial insurance in Ontario, because he stated that “I want to finally settle down, and be with someone exclusively and not have to worry about hooking up and stuff.” This account is notable for the ways in which this man’s evolving sexuality and intimacy needs are being expressed not strictly as a function of desire, but in relation to public policy that dictates the affordability of PrEP. This interviewee stopped using PrEP when his boyfriend told him to because they were monogamous, but then went back on PrEP after this boyfriend cheated on him. Another interviewee also discussed how his “monogamous” boyfriend “pressured” him to stop taking PrEP and then cheated on him. Like prior research, our participants discussed how a choice to stay on PrEP in a monogamous relationship may indicate that someone wishes to cheat (Felsher et al., 2021). These examples show how PrEP (dis)continuation is an interpersonally negotiated practice among GBQM, involving, beyond risk perception, a range of emotional and ethical dimensions, including conflicts and expectations within primary romantic partnerships.

Similar to other research on the PrEP Cascade (Newman et al., 2018), our data demonstrate that PrEP use is rarely linear and is highly contextual. Our participants discussed a more flexible relationship to PrEP, choosing to start, stop, pause, and restart as their sexual practices and relationships shifted, rather than taking it continuously and remaining “adherent.”

### Social Norms, Politics, and PrEP

Participants elaborated on how changing social norms informed their PrEP decision-making, including expectations to forgo condoms. One participant discussed how because of PrEP, “there are some people that just don’t want to use condoms and if you want to have sex with them, that’s kind of where you’re gonna be.” Others mentioned direct and indirect pressures to start or stop PrEP and/or to stop using condoms because of PrEP. One interviewee described seeing someone “casually” who was “very disappointed that I wasn’t on [PrEP],” while another mentioned having sexual partners that turned him down because he was not on PrEP and former “sexual partners in the past who are like, oh you *still* want to use condoms? So, there’s definitely been a shift.” One younger participant not on PrEP described how a hook-up who was on PrEP demanded that he have condomless sex:

We link up and then they tell me, ‘oh yeah, I prefer to bareback.’ [And I say,] that’s not fair, and that scares me. And then they tell me that I have no choice, it’s either you do it or you don’t [have sex]. If you’re asking me to risk myself or put myself at exposure, but they’re like, nothing will happen to you [because] I’m on PrEP.

This participant articulated how as a Black man, race and community played a vital role in his PrEP decision-making. He explained how his decision to not try PrEP was informed by perceived peer norms, “if my Black friends aren’t taking it then I’m obviously not going to take it.” This comment validates existing scholarship that demonstrates the significance of informal conversations about PrEP on increasing acceptability and interest within social networks (Felsher et al., 2021) and reflects Girard et al.’s (2019) assertions that PrEP may be creating different biosocialites among GBQM, forms of subjectivity and modes of belonging that are shaped in and through HIV technologies. This participant articulated how PrEP use among a cohort of Black men in his community made them “the desired ones. In our group we always have the one or two Black guys that are risky [by having more sex] because everyone wants them.” He mentioned how PrEP has allowed these men to start “catering to the white gays”: “Well some of them that are playing in that box [with white men], it’s like being a token as a Black man. You’re checking off boxes, right, for the masses.” This interviewee thus described PrEP as a form of social capital, as a way for some Black men to enter white socio-sexual networks containing problematic social dynamics.

A few participants described how some of their initial reservations about PrEP were rooted in social perceptions of PrEP users as “promiscuous” or “Truvada Whores” (Grace et al., 2018). As one interviewee declared, before he decided to take PrEP he was “completely judgemental” and wondered “how much of a whore can you be that you need PrEP?” However, another PrEP user described how these opinions have shifted, with PrEP moving from being stigmatized to becoming a marker of responsibility:

I would get guys saying, ‘no thanks, I’m not interested’ and I’d be like oh ok, and they’d be like, ‘well you’re on PrEP and I don’t want to hook up because you’re obviously on a whore rampage.’ That’s been said to me a few times. Now it’s like, I talk to guys who aren’t on PrEP and I’m like, ‘what the fuck, you’re not on PrEP, are you crazy?’

A few participants were critical of perceived increased social expectations for GBQM to be on PrEP. One man not taking PrEP stated “And this sort of push to get all gay men on PrEP is sort of like, because we’re somehow going to be, if we’re not [on PrEP], we’re sort of reckless queers, kind of thing.” In his view, PrEP is not necessary for him to be accountable to his sexual partners, community, and his sense of self as a queer man, especially because he is confident is his established safer sex strategies.

Also observing a heightened push to get all GBQM on PrEP, another interviewee stated

The pharmaceutical industry has made its bread and butter off the lives and deaths of gay men with this epidemic and now they’ve found out a way to broaden their base to not only those impacted directly by HIV, but also those of us who aren’t. Like, I was like, ah fuck, what a good business model: get all y’all motherfuckers on it!

This interviewee also expressed skepticism with how PrEP was being portrayed as a “panacea” for GBQM health despite deep-seated systemic and structural issues.

Another change in social norms discussed was the notion of serodifferent sex (i.e., sex between people with different HIV statuses) and serosorting (i.e., having sex with someone based on their HIV status), which have been long-standing issues in HIV prevention among GBQM (Kippax & Race, 2003). Participants mentioned becoming more comfortable having sex with men living with HIV if they were undetectable and no longer asking for the HIV status of new sexual partners because of PrEP. One participant stated, “I’m on PrEP, yeah I feel comfortable hooking up with undetectable people.” Another PrEP user discussed having sex with men living with HIV. Before PrEP, if a potential sex partner said that they were HIV-positive, he said “I just ghost them, even if that partner were undetectable.”

One interviewee not on PrEP mentioned how his anxieties with serodifferent sex have “dissipated significantly”:

I’ve had openly [HIV] pos partners [who are undetectable]. I’ve had all these things that are very different for me, that already was a fit for me. I already feel the effects in a PrEP present world that don’t necessarily require me to be on PrEP to benefit from it.

This interviewee articulated how it has become easier to navigate sex in the current biomedical moment with GBQM on PrEP or being undetectable. Though correct at understanding the “herd immunity” benefits of PrEP implementation, it is possible to question whether this participant’s trust in the *population* benefits of PrEP has created a false sense of security at the *individual* level.

Some PrEP users mentioned still “being hesitant” to have sex with men living with HIV. As one PrEP user articulated: “I’m still afraid of . . . like whenever I see ‘HIV-positive’ I know that it’s undetectable and I know that the chances are really low. Again, cause I read a lot and also doctors told me. But I don’t know.” Another interviewee also described not being “comfortable” having sex with HIV-positive men even though he is on PrEP. He said that this reflected “a bit of stigma” and an “irrational fear.” One PrEP user admitted that “it’s a little bit embarrassing to vocalize but I think I was still worried about having sex with people who were identifying as positive.” These participants’ self-critiques confirm prior research demonstrating growing social expectations for HIV-negative men to be more comfortable with serodifferent sex as a result of biomedical advances (Gaspar, 2019, Grace et al. 2020). Indeed, one PrEP user described how his decision to start PrEP was “almost like a political choice” because “the pressure is taken off of HIV-positive folks to always have to disclose or, you know, for some reason feel as though it’s their responsibility to worry about things like that.” Conversely, another interviewee on PrEP stated that PrEP and undetectability are creating new expectations on status disclosure that he did not agree with “Nowadays, guys who are [HIV-] positive don’t even feel like they need to tell you that they’re positive. [. . .] I’m just like, I don’t want to do it [have sex with HIV-positive men]. It’s my body I can do whatever the hell I want. It doesn’t mean I don’t understand all the facts.”

Although these social dynamics may at first appear distinct from the PrEP Cascade, they represent the social context fundamentally informing PrEP decision-making. Decisions to go on PrEP are highly motivated by changing norms and the influence of friends, peers, and sexual partners who may put pressure to start or stop PrEP, alter condom use, or have serodifferent sex.

### Growing up with Different Risks: Generations and the Life Course

Many participants explained their PrEP decision-making with reference to their generational cohort or age. Several mentioned growing up during the AIDS crisis years (80s and early 90s) and navigating sex before PrEP or “U=U.” Those older than 30 discussed living for decades with a deep-seated fear of HIV and gay sex. For some, these fears motivated their interest in PrEP. For example, one participant from the AIDS-2 Generation was “happy that there is PrEP . . . I did grow up with the idea that condomless sex is too risky,” while another man from the AIDS-1 generation discussed how being on PrEP gave him some “peace of mind.”

Conversely, one interviewee from the AIDS-1 Generation articulated how being sexually active in the 80s and being involved in AIDS activism informed his approach to safer sex that did not require PrEP:

My whole fear about contracting HIV is just not there. I don’t want to deliberately get HIV. It’s not a picnic having an ongoing health concern you have to take care of. There’s stigma associated with being HIV-positive. But the actual practical realities if I were [HIV] positive would not be catastrophic and a life ending kind of thing.

Confidence in seropositioning (i.e., only being the insertive anal sex partner if having sex with men living with HIV), along with concerns with costs, side-effects, and a diminishing libido associated with aging, led this participant to conclude that PrEP was not a worthwhile option.

One man from the AIDS-2 Generation expressed ambivalence toward PrEP:

I’m [in my 40s]. So when I was like 20, like there wasn’t, you know, HIV was still like a death sentence kind of a thing. So, these kinds of things [like PrEP] are exciting, but I guess I yea . . . I’m not sure, I don’t know what my hesitation is in terms of wanting to take it fully or know more about it.

For some GBQM, PrEP hesitancy (including the initial cynicism of eventual PrEP adopters) was shaped by strong symbolic and affective attachments to previous notions of *safer sex*, and in particular, the necessity of condoms. For example, one interviewee currently on PrEP discussed how “I always assumed that not using a condom equated to AIDS.” Another PrEP user spoke about generational attachments to condoms: “I think guys in my age group have like more of a fear of contracting HIV than the guys now who are 20 who are like, what are condoms?”

A participant from the AIDS-2 generation articulated his “reluctance” to try PrEP after years of complying with public health measures: “Particularly [for] my generation, we’ve been raised with that safe sex discourse which is a very draconian and top down and scary. So sometimes, it’s those remnants where I feel my own reluctance [to try PrEP].” Another interviewee from the same generation who decided he did not need to take PrEP described his strong attachments to older notions of safer sex (i.e., condoms) and feeling worried that PrEP would derail years of community work trying to get GBQM to use condoms: “So now to say, yo, take this pill and don’t even think about it. I was like, ugh, fuck, all of those years of behaviour change work sort of scuttled out in one moment.”

This participant articulated how his initial criticism was based on working from “an older model” of sexual health and was motivated by envy toward younger GBQM who he saw as no longer having to manage the HIV-related anxieties he had when growing up:

My identity as a queer person, a queer man, was so interlinked with the looming threat of HIV that whether I was conscious of it or not, my early reticence and skepticism around these other options [like PrEP] was almost comparable to that example of an old person going, ‘oh well, I grew up it was really hard and these [younger] motherfuckers have it too easy.’ Well we all should have it a little bit easier, right? And so yea, I was like, we should all be free of this [fear of HIV].

This participant went on to consider how different his life could have been if PrEP were available to him at a younger age:

What would it have been like had I come up in a different era where I could have explored my identity and sexuality without the constant fear? How might I have been and acted different? What other choices might I have made? I often think about experiences and allowances that I didn’t give myself growing up from 14 onwards that I just would have maybe chosen differently had I not been always afraid [of HIV].

In contrast, a PrEP user from the AIDS-1 generation discussed “feeling good about how it is nicer and better for the younger generation now.” He articulated how advances like PrEP “makes me happy” because compared with his youth when “AIDS, HIV, was in full swing, you really didn’t want to talk about your sexuality at all because . . . well, whatever, you were just gonna die anyway.” Other participants over 30 years old also noted how different they thought sex and queer life was for younger GBQM because of PrEP. One interviewee on PrEP for example, observed that “younger generations are a little more at ease about their bodies, their sexual lives” and more open to “disclosing HIV status, or talking about STIs just over brunch.”

Participants younger than 30 (the PrEP and “U=U” generation) often described a different relationship to sex than older men because of HIV biomedical advances. For example, one younger interviewee stated that “I’ve not had a lot of anxiety around [HIV] transmission, I think part of that was because of that . . . I think more recent medical advancements in the treatment of HIV.” PrEP in particular has allowed some younger GBQM to have sex with comparatively less fear than their older counterparts. One younger interviewee, for example, stated that he would be on PrEP “for the rest of my life.” He described immigrating to Canada from a country where homosexuality is illegal and having lots of queer sex was framed as being directly tied to PrEP. PrEP was a fundamental aspect of establishing his queer identity and sexual freedoms.

In summary, participants’ evaluation of their eligibility and need for PrEP, the first step in the PrEP Cascade, was interlinked with their perceptions on previous safer sex strategies like condoms and serosorting. This went beyond a biomedical assessment comparing PrEP use to condom use, to making sense of the deeply entrenched meanings and affective dimensions of long-established safer sex strategies.

## Discussion

Compared to earlier qualitative research on new HIV technologies in the Canadian context (Klassen et al., 2017), our investigation demonstrated a high level of awareness and the normalization of its use among GBQM, showing that implementation efforts have been successful at shifting PrEP from being a minor, somewhat exclusive prevention option for a few well networked GBQM, to a commonplace prevention strategy. While social and cultural factors, preferences for established prevention methods, and risk perception greatly determined how GBQM evaluated whether PrEP was useful or not, the participants’ account demonstrated that rather than beliefs about PrEP being set in stone, GBQM are engaging in an ongoing reflexive process and changing their PrEP opinions and strategies over time. The affordability of PrEP remained the biggest barrier to access and affected perceptions on sexual desire and HIV risk.

**Figure 1.**
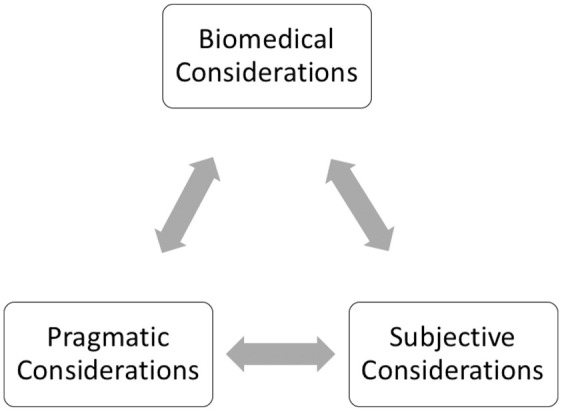
Reflexive PrEP Decision-Making

PrEP decision-making is more complex than a sequential passing through a linear PrEP Cascade (Newman et al., 2018). GBQM choose to start, stop, pause, restart, and end PrEP use in relation to shifting pragmatic considerations (logistical effort, financial costs, systemic barriers), biomedical considerations (efficacy, side-effects, and STIs) and subjective considerations (generational attachments, identity, community politics, and changing sexual preferences). As noted in Figure 1, these three components mutually inform one another causing GBQM to (re)evaluate the utility of PrEP at a particular moment in their life course and in particular social contexts. Neither set of considerations can be understood without reference to the other. A GBQM’s sex life cannot change due to PrEP if he or his partners cannot afford to get it. Someone’s ability to pay for PrEP is inconsequential if their concern with side-effects prevents them from trying it. Someone’s worries regarding the potential risk of PrEP side-effects can shift based on his evolving perceptions of PrEP’s benefit to his sex life. And these perceptions can also depend on whether he sees the work of accessing PrEP as easy or cumbersome.

In terms of pragmatic considerations, the clear policy imperative, especially in Ontario where the data indicate that cost is the most significant barrier to PrEP initiation, is to make PrEP free for all groups who meet clinical guidelines. At a broader structural level, such data also speak to the benefits of implementing a robust national pharmacare/drug plan for all Canadians (Gaspar et al., 2021).

In terms of biomedical considerations, concerns with longer-term side-effects (i.e., organ damage) can be deterrents to trying PrEP (Philpot et al., 2020). While such side-effects are rare and manageable, they do represent valid concerns that must be monitored if one takes PrEP. With these side-effects in mind, if a GBQM is not consistently at risk for HIV—that is, if he is not sexually active for sustained periods of time—it may be beneficial for him to consider the benefits of on-demand or episodic regimens of PrEP rather than continuous use. More communication about the manageability of long-term side-effects and how these should be factored into PrEP decision-making may help to get some GBQM more comfortable with PrEP. Existing community resources make short mention of such side-effects (Freddie, 2022; GMSH, 2021; HIM, 2022; Ontario PrEP Start, 2022). And as one participant mentioned, there are also fewer resources available that detail how to take PrEP on-demand or episodically and how to pause PrEP safely. More resources about taking PrEP in a flexible fashion are needed to respond to the realities of how GBQM are actually using PrEP in real world contexts. It is important, however, to distinguish when GBQM are taking PrEP in a flexible fashion because of desired changes to their sexual practices versus when GBQM are forced to stop PrEP because a change in their employment status affects their ability to pay for it.

For subjective considerations, PrEP’s availability offers an opportunity for GBQM to adjust their sexual practices. Like Newman et al. (2018), our analysis indicates that upholding strict analytical distinctions between PrEP users from non-PrEP users is limiting, as both shared similar sexual behaviors. It was their subjective perceptions of HIV risk in this new biomedical context that are different, perceptions that were relational and context dependent.

For example, thinking about HIV and AIDS through a generational lens was common in the sample. Like Hammack et al. (2019), we observed that a hesitancy to PrEP tended to be found more among men in the AIDS-2 generation (men born in the 80s and 90s). However, some from the AIDS-1 generation (men who were of sexual age during the 80s) also reported a hesitancy toward PrEP. Many participants situated PrEP within a larger historical context to make sense of how “safer sex” was changing. Strong symbolic and affective attachments to established versions of safer sex (condoms and/or serosorting), and decades of fear-based messaging regarding HIV have made some GBQM reluctant to try PrEP (Gaspar, 2017), even though they believe that PrEP works and is beneficial for other sexually active GBQM. It is also evident that younger GBQM (“zoomers,” the youngest “millennials,” or the PrEP and “U=U” generation) are perceived to be growing up in a different sexual landscape, one less saturated by fears and concerns of HIV.

In some cases, a GBQM’s history of being compliant with previous public health advice made them resistant to trying PrEP, as they could not imagine abandoning condoms with casual partners or having sex with men living with HIV. Even when knowledge and technologies shift, some people may be unwilling to change their behaviors (Gaspar, 2019). In other cases, GBQM’s critiques of needing to be compliant with public health rules, and a distrust with the corporate interests behind PrEP, made them reluctant to try PrEP (Young et al., 2016). These men resisted the notion that GBQM are ‘reckless’ hedonists in need of biomedical interventions.

PrEP decision-making cannot be reduced to generational categories, as our sample demonstrates that there are not uniform beliefs among men within the same generational cohort. Nonetheless, these categories do help to explain how the ways in which HIV risk and sexual identity are linked depend on how one’s life course has intersected with various historical moments within the epidemic. For older men, a dis/interest in PrEP was not *just* a result of considering one’s biological risk for HIV, but was shaped in and through an assessment of how one has been socially and psychologically impacted by navigating the epidemic for decades. These psychosocial generational dynamics can also help to explain why some lower risk men are sometimes interested in taking PrEP—that is, as a reprieve from long-standing anxiety. At the level of health promotion, PrEP messaging may eventually need to respond to these generational differences. This will especially be true if younger people’s relationship to the HIV epidemic becomes gradually dissimilar from the lived experienced of their older peers, particularly if biomedical advances are successful at eliminating (or at least significantly reducing) new HIV infections. This last point also raises important sociological questions. It is not new for GBQM’s sexual practices, identity, and community politics to shift in response to technological advances, and for such advances to have differing effects across generations (Hammack, 2019; Race, 2014). However, the idea that these shifts may lead us (close) to the end of epidemic and can help to eliminate the fears and anxiety long associated with queer sex because of HIV, will have profound social implications. The participants’ accounts above already demonstrate potentials for some resentment toward younger men over the freedoms afforded by this new biomedical moment, and an inability for some to “let go” of previous narratives held about HIV and queer sex. What will queer identity and sexual practices look like when HIV is no longer the omnipresent force that it has been for decades? Will it be possible or even politically desirable to untangle queer identity from HIV? Who might resist this change and why?

Our work confirms the extant literature that demonstrates that PrEP may promote varied sexual practices in terms of condomless sex or increases in sexual activity (Grov et al., 2021). HIV prevention is not just a set of behaviors or habits shaped by “risk compensation,” but is, as several participants explained, fundamental to developing a sense of self, community belonging, and developing sexual agency (García-Iglesias, 2020; Gaspar, 2017; Girard et al., 2019; Grov et al., 2021; Skinta et al., 2020; Williamson et al., 2019). Opportunities for increased forms of sexual agency and pleasure are undoubtedly a positive result of PrEP rollout. However, some of the ethical, emotional, and social dynamics that may arise as GBQM navigate a “PrEP present world” demonstrate that there may be some moments of intra and interpersonal conflict, and as noted above, possible tensions across generational lines. PrEP offers an opportunity for GBQM (with access) to re-imagine and re-evaluate their sexual pasts, futures, and identity, by ontologically changing what sex is (Race, 2014). The data strongly demonstrate that exactly how PrEP availability comes to affect sexual practice and sexual identity can be quite diverse among GBQM. There is no one way in which GBQM are taking up (or avoiding taking up) PrEP. Our analysis indicates that how GBQM use or avoid PrEP is often personal, complex, and dynamic. Motivations for starting, stopping, pausing, and restarting PrEP are as innumerable as the number of sexual possibilities a GBQM faces. When a participant asked “What other choices might I have made?” he was reflecting on how PrEP could have altered the course of his sexual history and identity. However, beyond reconsidering past behavior, this query reflects the *ongoing reflexivity* of evaluating his sexual choices at new junctures in his life.

Many of the social and cultural dynamics explored above have parallels with research findings coming from the UK (Young et al., 2016), US (Hammack et al., 2019), and Australian (Philpot et al., 2020) contexts. However, there are regional differences in policies and program delivery that affect whether GBQM can afford PrEP. For instance, the NHS in the UK currently offers PrEP free of charge. Even *within* Canada, comparing the perspectives of GBQM in BC (where PrEP is publicly covered for all) to Ontario (where PrEP is not), demonstrates how financial barriers affect perceptions of the social and personal significance of PrEP. Compared to BC, the sample in Ontario expressed somewhat more skepticism toward PrEP. Not being able to afford PrEP can lead some people to think that it is less necessary or less helpful for them, regardless of what their sexual risk-taking may be. The implementation of PrEP services needs to respond to local contexts as no one size fits all. Our participants looked for information and resources from *local* community agencies and clinics. However, it is also important to respond to the inequitable access to PrEP that exists both within rich Western nations and in the Global South, if PrEP is truly going to be a tool used to end the HIV epidemic.

Examining PrEP implementation in the US context, Golub and Myers (2019) state that an emphasis on risk perception will not be enough to increase uptake. This argument is based on evidence indicating that many GBQM may be inaccurately evaluating their objective risks for HIV. We would frame this somewhat differently. Our analysis indicates that the utility of PrEP is being read in and through many complex social considerations that then inform how people interpret risk. As such, it may not be that GBQM are calculating risk “incorrectly,” but that information and assessment tools for PrEP must move beyond strict bio-behavioral calculations of risk, to better incorporate some the subjective dimensions outlined in this paper which can help GBQM make informed decisions.

Finally, considering all these complex and emergent social expectations in the “PrEP present world,” how can healthcare providers and health promotion specialists ensure that they are empowering GBQM in their PrEP and sexual decision-making throughout the Cascade? The data suggest that targeted sexual health counseling, at least for some GBQM, may be key to facilitating effective implementation of PrEP and of reducing risks associated with PrEP use, especially from pauses in use and abrupt discontinuation. Resource constraints may limit the amount of time that healthcare providers have to explore these issues in depth with patients at each visit. Nonetheless, sexual health counseling remains key. Like other studies that have argued for the need to integrate mental health services with sexual health and social services (Lacombe-Duncan et al., 2021; Salway et al., 2019), our analysis indicates that opportunities for GBQM to discuss their changing sexual health needs (including shifts in their romantic and intimate partner relationships) with trained professionals (including peer counselors), may help some GBQM make more informed decisions about PrEP. Our analysis thus substantiates the need for more resources to be directed to sexual health counselors and sexual health service providers. As part of this counseling, providers must recognize that daily PrEP may not work for all, and should be more willing to look at on-demand PrEP. However, the limited use of on-demand PrEP in Canada and its current status as an “off-label” regime (not fully authorized by Health Canada), may make some GBQM and providers reluctant to pursue it as an option.

Beyond individual-level counseling, community programming aimed at helping GBQM navigate potential conflicts caused by a shifting biomedical and sexual landscape, could be useful by empowering GBQM in their sexual decision-making. These may take the form of creative workshops, podcasts, online group sessions, and community art events that allow for GBQM to share their evolving experiences with new HIV technologies (PrEP and U=U). GBQM need clear lines of communication and trust with healthcare providers to talk about PrEP.

This study is limited by focusing on GBQM who live in or by a major urban center. Additional research on PrEP use in more remote and rural regions is needed. Our sample fell short of representing all groups, such as Indigenous representation in Ontario, and trans representation in BC. We did not speak to people who identified as sex workers, those who have experienced homelessness or who identified as people who use injection drugs. As we recruited through our community partners and associated clinics, our sample may over represent GBQM well networked with HIV health agencies and whose baseline knowledge of and ability to access PrEP is higher, a persistent methodological limitation in HIV community-based research (Gaspar, 2019). The analysis above is also only cross-sectional. While the interviews were mostly conducted virtually due to COVID-19, the richness of the transcripts and the enthusiasm of participants does not indicate that the data quality was negatively affected by the online format.

## Conclusion

In summary, the PrEP Cascade has been generated to help understand where barriers arise at various points in the PrEP decision-making process. In addition to pragmatic barriers and biomedical concerns, PrEP has significant social implications with its uptake altering sexual practices and sexuality in diverse and complex ways. The more healthcare providers and health promotion experts are able to understand these shifts in sexuality, the better equipped they will be at producing education, soliciting questions, and refining their messages to clients to ensure that GBQM are making informed choices about PrEP that effectively reduce their risks.
